# Individualized, cross‐validated prediction of future dementia using cognitive assessments in people with mild cognitive symptoms

**DOI:** 10.1002/alz.14305

**Published:** 2024-10-17

**Authors:** Emma Borland, Niklas Mattson‐Carlgren, Pontus Tideman, the Alzheimer's Disease Neuroimaging Initiative, Erik Stomrud, Oskar Hansson, Sebastian Palmqvist

**Affiliations:** ^1^ Clinical Memory Research Unit Department of Clinical Sciences, Malmö Faculty of Medicine at Lund University Clinical Research Centre Malmö Sweden; ^2^ Department of Neurology Skåne University Hospital Malmö Sweden; ^3^ Wallenberg Center for Molecular Medicine Lund University Lund Sweden; ^4^ Memory Clinic Skåne University Hospital Malmö Sweden

**Keywords:** cognitive impairment, dementia, mild cognitive impairment, prediction dementia, subjective cognitive decline, two‐step model

## Abstract

**INTRODUCTION:**

We aimed to develop an algorithm to predict the individualized risk of future dementia using brief cognitive tests suitable for primary care.

**METHODS:**

We included 612 participants with subjective cognitive decline (SCD) or mild cognitive impairment (MCI) from the Alzheimer's Disease Neuroimaging Initiative (ADNI) study, assessed for at least 4 years or until progression to dementia. A logistic regression model, using cognitive tests as predictors and dementia progression as an outcome, stratified participants into low, intermediate, or high risk. A second model, including 1‐year cognitive test changes, was applied to the intermediate group. The models were replicated in 392 SCD/MCI participants from the BioFINDER‐1 study.

**RESULTS:**

The best two‐step model for predicting dementia incorporated Trail Making Test B (attention/executive function), Animal Fluency (verbal fluency), Mini‐Mental State Examination (global cognition), and 10‐word list recall (memory). The model's positive predictive value in ADNI was 85.8% and negative predictive value was 92.2% versus 62.5% and 95.6%, respectively, in BioFINDER‐1.

**DISCUSSION:**

This two‐step model accurately predicts individualized dementia risk.

**Highlights:**

- To our knowledge, this is the first algorithm for predicting all‐cause dementia using a novel two‐step model utilizing brief cognitive tests.
- Applying a validated model including the Trail Making Test B, Animal Fluency, MMSE, Alzheimer's Disease Assessment Scale delayed, and immediate recall can robustly and accurately categorize individuals into low, intermediate, or high risk of dementia progression and can facilitate clinical decision‐making and personalized patient care.
- We created an app that is available for research and educational purposes at https://brainapps.shinyapps.io/PredictAllCauseDementia to provide an individualized risk score for dementia progression.

## 1 BACKGROUND

Dementia, or major neurocognitive disorder, is a clinical syndrome characterized by changes in cognition and behavior as well as impairments of activities in daily living.^1^ The syndrome affects more than 50 million people worldwide and is anticipated to double by 2050.^2^ The most common cause of dementia is Alzheimer's disease (AD), while less common causes are vascular disease, Parkinson's disease, dementia with Lewy bodies (DLB), and frontotemporal dementia (FTD).^3^, ^4^ Regardless of whether the treatment is focused on lifestyle and multimodal interventions or novel anti‐amyloid treatment for AD, it is hypothesized that such interventions should be started as early as possible.^5^, ^6^, ^7^ It is therefore crucial to optimize the early diagnostic process. Though there are now available blood‐based biomarkers, such as phospho‐tau217 and neurofilament light for early detection of neurocognitive disorders,^8^, ^9^, ^10^, ^11^ they are not widely available for diagnostic workup or screening. Further, the best performing biomarkers are primarily studied for assessing AD. However, the diagnostic landscape for cognitive impairment is highly diverse, encompassing various cognitive disorders that may eventually lead to dementia.

There is already approved anti‐amyloid treatment for AD in the United States and elsewhere.^12^ With available disease‐modifying treatment for AD, there will expectantly be a significant increase in the number of patients seeking healthcare for evaluation of their cognitive symptoms. Therefore, in everyday clinical practice, clinicians urgently need accessible and brief decision‐support algorithms, especially in primary care, where the triaging of patients with cognitive impairment first takes place. These algorithms could help identify two groups: patients at high risk of progressing to dementia, who require further evaluation to determine the underlying cause, and those at low risk, who can either be reassured or monitored in primary care, with follow‐up risk assessments to optimize symptom management and plan effectively for the future.^13^

Although previous studies investigated predictors of future dementia,^2^, ^14^, ^15^, ^16^, ^17^, ^18^, ^19^, ^20^, ^21^, ^22^ none developed algorithms utilizing easily accessible measures for individualized prognosis in individuals seeking care for cognitive symptoms. Furthermore, these studies do not reflect a real clinical setting where people with subjective or objective cognitive symptoms seek healthcare. External validation of existing dementia prediction models has highlighted the need for updated models due to high variability in discriminative power (area under the curve [AUC] ranging from 0.55 to 0.81).^23^ Repeated measures over time allow for dynamic prediction models that can be updated as new information becomes available. Previous research suggested that multiple serial measurements over time can enhance the predictive accuracy of AD.^24^, ^25^, ^26^ However, to our knowledge, no previous models created practical tools for clinicians to predict individual progression to dementia based on longitudinal cognitive test results.

Patients seek healthcare at various stages of their cognitive decline, ranging from having subtle subjective symptoms to clear objective impairment.^27^ Some individuals may present with an evident low or high risk of progressing to dementia based on the initial assessment. However, for other patients, the initial evaluation may be inconclusive, necessitating follow‐up with repeated testing. Such a two‐step workflow has been applied in the screening for AD using plasma biomarkers and proved to effectively reduce the need for confirmatory testing in some cases.^28^ Thus, we hypothesized that a two‐step model for cognitive testing would align well with clinical practice and current workflows. We aimed to develop a model for predicting all‐cause dementia at 4 years by analyzing existing longitudinal data in individuals with cognitive symptoms from the Alzheimer's Disease Neuroimaging Initiative (ADNI) study. We planned to replicate our findings in the BioFINDER‐1 study. We also aimed to develop an app that calculates individualized risk scores.

**RESEARCH IN CONTEXT**

1. **Systematic review**: We reviewed the literature using PubMed. We found no previous studies for individualized prediction of all‐cause dementia based on data from people with subjective cognitive decline or mild cognitive impairment using brief cognitive tests.
2. **Interpretation**: In a cohort from the Alzheimer's Disease Neuroimaging Initiative (ADNI) study, a logistic regression model was trained using baseline brief cognitive tests and progression to dementia as outcome to identify those at low, intermediate, and high risk of future dementia, at 4 years from baseline. A second model, including additional follow‐up tests at 1 year from baseline, was trained in those at intermediate risk. We replicated the two‐step model in the BioFINDER‐1 study, where it had a high total negative predictive value (95.6%) and a moderate positive predictive value (62.5%). This approach could, for example, assist primary care physicians in developing a patient care plan based on the initial visit. For intermediate‐risk patients, it may involve scheduling follow‐up cognitive testing a year later, while high‐risk patients could be referred to secondary care for further evaluation. For low‐risk patients, the focus could shift to identifying other possible causes of cognitive symptoms, such as psychiatric disorders or medication side effects. An app was developed for calculating the individualized risk for progression to all‐cause dementia using these models (https://brainapps.shinyapps.io/PredictAllCauseDementia).
3. **Future directions**: Further studies should validate the models on larger, more diverse populations. Additional tests, including novel digital cognitive tests, could improve their accuracy.

## 2 METHODS

### 2.1 Participants

**FIGURE 1:**
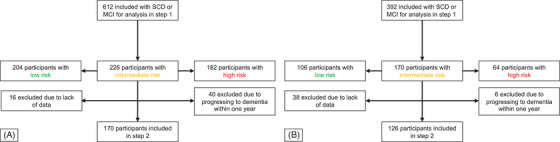
Selection of participants and numbers at each step in ADNI (A) and in BioFINDER‐1 (B).

In Table 1, we present the demographics of the participants. For the training cohort, where the models were developed, we included 612 participants with subjective cognitive disorder (SCD) and with mild cognitive impairment (MCI) from ADNI1, ADNI2, and ADNI3 within the prospective and longitudinal ADNI study (https://adni.loni.usc.edu/). All participants from ADNI had complete data for all the examined cognitive assessments. The models, with cutoffs established using the training cohort, were then applied to a validation cohort from the prospective and longitudinal BioFINDER‐1 study (NCT01208675**)**. Participants with cognitive complaints were included consecutively in BioFINDER‐1 and were categorized as having SCD or MCI. We included 176 participants from BioFINDER‐1 with SCD and 216 with MCI, all of whom were followed for at least 4 years or until they progressed to dementia (AD or other dementia types) within that timeframe, according to our previously published[Table alz14305-tbl-0001] methodology.^8^ Figure 1 describes the selection of participants in ADNI and BioFINDER‐1 at the different steps. Inclusion and exclusion criteria for ADNI and BioFINDER‐1 studies are outlined in Supplementary File 1. We chose the ADNI dataset as a training study due to its larger cohort size (612 participants compared to 392 in BioFINDER‐1). Additionally, the BioFINDER‐1 cohort more closely mirrors real‐world scenarios, as study participants were recruited from a memory clinic while seeking help for cognitive symptoms.

**TABLE 1 alz14305-tbl-0001:** Demographics for participants in ADNI and BioFINDER‐1.

	ADNI			BioFINDER‐1		
	Progressing to dementia	Not progressing	*p*	Progressing to dementia	Not progressing	*p*
No. participants included at baseline	7 SCD, 294 MCI	103 SCD, 208 MCI	<0.001	24 SCD, 117 MCI	152 SCD, 99 MCI	<0.001
No. participants included at 1‐year follow up	0 SCD, 76 MCI	5 SCD, 89 MCI	0.1	12 SCD, 26 MCI	48 SCD, 51 MCI	0.09
Education, years (SD)	16.0 (2.8)	16.5 (2.6)	<0.001	11.3 (3.4)	12.0 (3.5)	0.06
Age (SD)	74.1 (7.1)	71.8 (6.3)	<0.001	72.2 (5.2)	70.2 (5.5)	<0.05
Sex	124 female, 150 male	177 female, 161 male	0.1	80 Male, 61 Female	122 Male, 129 Female	0.14
Tests at baseline (mean, SD)						
MMSE	26.9 (1.8)	28.8 (1.4)	<0.001	27.1 (1.8)	28.1 (1.7)	<0.001
ADAS delayed recall	7.0 (2.2)	3.4 (2.2)	<0.001	7.0 (2.0)	4.0 (2.4)	<0.001
ADAS immediate	5.9 (1.6)	3.6 (1.5)	<0.001	5.4 (1.2)	3.7 (1.5)	<0.001
Animal fluency	15.2 (4.6)	20.4 (5.1)	<0.001	13.0 (5.0)	19.3 (6.0)	<0.001
TMTA	47.9 (23.6)	33.2 (11.7)	<0.001	63.3 (23.0)	46.8 (16.5)	<0.001
TMTB	144.4 (78.0)	79.9 (35.3)	<0.001	133.4 (29.5)	104.7 (27.6)	<0.001

Abbreviations: ADAS, Alzheimer's Disease Assessment Scale; BL, baseline; MCI, mild cognitive impairment; MMSE, Mini‐Mental State Examination; SCD, subjective cognitive decline; SD, standard deviation; TMTA, Trail Making Test A; TMTB, Trail Making Test B.

### 2.2 Cognitive tests and other predictors of future dementia

The cognitive tests included in the model selection were the Alzheimer's Disease Assessment Scale (ADAS) immediate recall, ADAS delayed word recall, Naming Objects and Fingers and Constructional Praxis parts from ADAS‐cog, Animal Fluency, Mini‐Mental State Examination (MMSE), Trail Making Test A and B (TMTA and TMTB), Clock Drawing Test, and Copy Clock Test. In ADAS immediate recall, participants are given 10 words printed on white cards and are asked to read the words aloud and remember them. Participants then attempt to recall as many words as possible from the list. The procedure is repeated for two additional learning and recall trials. The ADAS immediate recall score is the average of the three learning trials. The ADAS delayed recall trial is the number of words remembered after a short delay. For both the ADAS delayed recall and ADAS immediate recall subscales, points are counted as the number of words the participant does not recall, with higher points indicating worse performance.^29^ In the Naming Objects and Fingers subscale of ADAS, participants are asked to name 12 randomly presented real objects, which vary in frequency from high to low, as well as name the fingers on his/her dominant hand. In the Constructional Praxis subscale, participants are asked to copy four geometric forms, ranging from a simple circle to a more complex cube.^30^ In Animal Fluency, participants are asked to name as many animals as they can in 60 s.^31^ The MMSE is a widely recognized global cognitive assessment with a maximum score of 30 points.^32^ In TMTA, participants draw lines between numbers in ascending order. In TMTB, participants alternate between letters and numbers in the correct order. The result is the time it takes participants to complete the tests. TMTA and TMTB assess visual scanning, processing speed, and mental flexibility.^33^ In the BioFINDER‐1 study, TMTB was performed every other year. Therefore, the TMTB results at the 1‐year follow‐up were calculated by taking the difference in baseline and 2‐year follow‐up results divided by 2 to determine a mean annual change. In the Clock Drawing Test, participants are asked to draw a clock, fill in all the numbers, and set the hands for 10 past 11. Following this, participants are asked to copy a clock from a model, referred here to as the Copy Clock Test. The Clock Drawing Test assesses auditory comprehension, planning, visual memory and reconstruction, visuospatial abilities, and motor programming and execution.^34^

Other predictors included in the model were sex, age, and years of education. Since magnetic resonance imaging (MRI) is often included in the initial work‐up, we included cortical thickness from temporal regions (which are prone to atrophy caused by AD^35^, ^36^) in a secondary analysis. Cortical thickness was quantified using FreeSurfer version 5.1 (http://surfer.nmr.mgh.harvard.edu). The temporal composite was calculated based on the cortical thickness in the entorhinal, inferior temporal, middle temporal, and fusiform regions, as described elsewhere.^37^ In the ADNI study, MRI scans were conducted with 1.5T or 3T scanners, and for this study we used non‐accelerated T1 images. All participants from BioFINDER‐1 were imaged with a 3T Siemens Trio Scanner.

### 2.3 Outcome

The outcome variable was progression to any dementia within 4 years. In the ADNI study, participants undergo a series of tests repeated at intervals over subsequent years, including clinical evaluation, neuropsychological testing, genetic testing, lumbar puncture, MRI, and positron emission tomography (PET) scans. Based on these measures, a physician determines progression to dementia (see study protocols for ADNI at https://adni.loni.usc.edu/methods/documents/). In BioFINDER‐1, dementia was diagnosed through clinical consensus assessments according to the Diagnostic and Statistical Manual of Mental Disorders, Fifth Edition (DSM‐5) criteria for major neurocognitive disorders^38^ by physicians at the Memory Clinic, as previously described.^8^

### 2.4 Statistics

Logistic regression and receiver operating characteristic (ROC) analyses were used to predict future dementia. The outcome variable was progression to dementia within 4 years (yes/no, ie, presence of dementia at 4 years). The predictors examined in the first step were age at baseline, sex, years of education, and total scores for the cognitive assessments MMSE, ADAS delayed recall, ADAS immediate recall, ADAS Naming Objects and Fingers, ADAS Constructional Praxis, Clock Drawing Test, Copy Clock Test, TMTA, TMTB, and Animal Fluency. Model selection was data‐driven, using the MuMIn package (Multi‐model Inference) in R. This approach tested all possible predictor combinations to identify the model with the lowest Akaike information criterion (AIC) and the fewest predictors, as previously described.^39^ If several were found, the model with the fewest variables was chosen to keep the model as parsimonious as possible. Using the probability output from this logistic regression model, we applied thresholds to define high and low probability (of progression to dementia). These were set at 95% specificity and 95% sensitivity, following a previously described stratification method.^40^, ^41^ To verify that the model was not overfitted due to the many combinations tried, we utilized an independent replication cohort for validating the model.

For participants with intermediate risk (probabilities between the cutoffs for high and low probability), a second model selection was performed, now also including 1‐year changes in test results. Figure 1 describes the selection of participants in ADNI and BioFINDER‐1 for the different steps. The probability cutoff for low risk of progression to dementia in this second step was set at 90% sensitivity to ensure a high negative predictive value (NPV), which is important in an initial work‐up in primary care to rule out underlying dementia. Note that this second step only included one cutoff, thereby eliminating the intermediate risk. All statistical analyses were performed using the R programming language (version 4.2.2). *p* values for comparisons between ADNI and BioFINDER‐1 and between progressing versus non‐progressing groups were calculated using a *t* test for age and education, Fisher's exact test for sex, progression to dementia, and SCD/MCI and Spearman's rank correlation test for associations between test results. Additionally, we created an online tool for predicting an individual's risk of progression to all‐cause dementia within 4 years by inserting results from the logistic regression models.

## 3 RESULTS

### 3.1 Demographics

We included 612 participants from the ADNI study who were classified as either SCD or MCI. The mean age of the total cohort was 72.9 years (SD 6.8), 338 (55.2%) were men, and the mean number of education years was 16.3 (SD 2.7). Out of these participants, 301 (49.2%) progressed to dementia within 4 years. We replicated our findings in 392 participants from the BioFINDER‐1 study. The mean age for the total cohort was 70.9 years (SD 5.5), 202 (51.5%) were men, and the mean level of education was 11.8 (SD 3.5) years. A total of 141 (36.0%) participants in BioFINDER‐1 progressed to dementia within 4 years (see Table 1 for demographics). Comparisons of demographic factors between ADNI and BioFINDER‐1 are detailed in Table S1. In BioFINDER‐1, 298 (76.0%) were referred to the Memory Clinic from primary care and 37 (9.4%) from other specialist clinics, and 32 (8.2%) were self‐referrals. Referral method data were missing for 25 participants (6.4%). The participants were followed for an average of 5.2 years (SD 2.8). Among the 141 who progressed to dementia within 4 years, the mean time to progression was 3.4 years (SD 2.1). Additionally, 80 participants progressed to dementia later than the 4‐year follow‐up period, doing so within a mean of 5.8 (SD 1.4) years. The 171 participants who did not progress to dementia were followed for a mean of 7.4 years (mean 1.7).

### 3.2 First model selection step in training cohort (ADNI)

**FIGURE 2:**
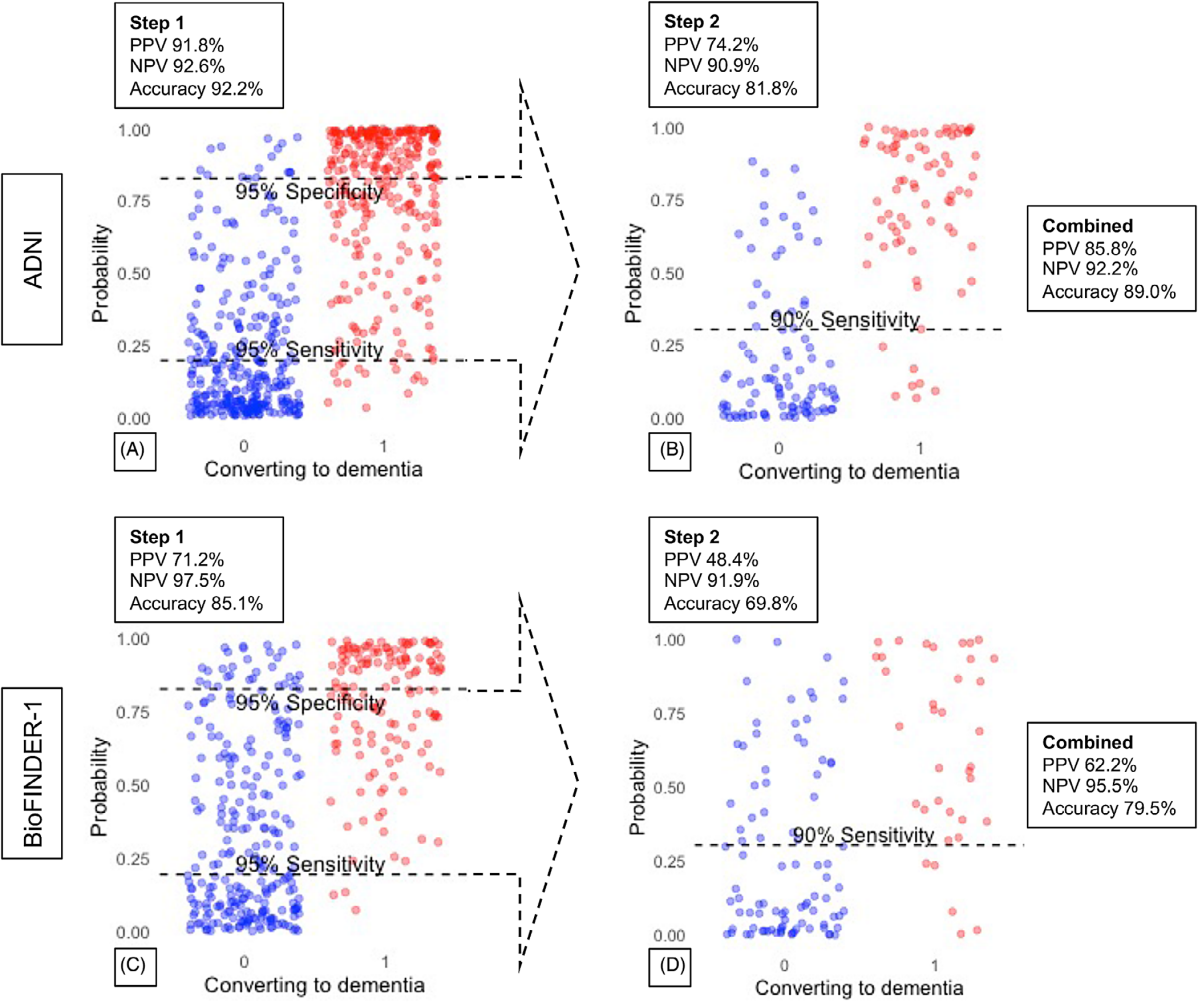
Two‐step model predicting progression to dementia in ADNI and BioFINDER‐1. (A) Probabilities of progressing to dementia within 4 years in Step 1 according to model (*N* = 612). (B) Intermediate group shows probability of progressing to dementia within 3 years (*N* = 170). (C) Probabilities of progressing to dementia within 4 years in BioFINDER‐1 using Step 1 model and cutoffs derived in ADNI (*N* = 392). (D) Probabilities of progressing to dementia within 3 years in intermediate group in BioFINDER‐1 using Step 2 model and cutoff derived in ADNI (*N* = 126).

In the first step (Figure 2A), the MuMIn model identified the best model for predicting progression to dementia within 4 years in ADNI based on the lowest AIC. The best model included the following variables: ADAS delayed recall, ADAS immediate recall, Animal Fluency, TMTA, and TMTB (Supplementary File 2A). None of the demographic variables, age, sex, or education, were selected using the MuMIn package in R. All remaining variables were statistically significant (*p* < .05). The final model gave an AUC of 0.91 (95% confidence interval [CI]: 0.89 to 0.94) in ADNI. When applying upper and lower cutoffs at 95% sensitivity and 95% specificity from the logistic regression model, the resulting positive predictive value (PPV) was 91.8% (with 29.7% of subjects above the upper cutoff), the NPV was 92.6% (with 33.3% of subjects below the lower cutoff), and accuracy was 92.2% (excluding participants with an intermediate probability between the cutoffs, which comprised 36.9% of subjects). A total of 122 participants in ADNI progressed to dementia within 1 year, five of these were screened as being at low risk, 40 as being at intermediate risk, and 77 as being at high risk at the first step. We present baseline test results for these individuals in Table S2. Among the 110 participants with SCD, 87 (79.1%) were classified as low risk for progression to dementia, two (1.8%) as high risk, and 21 (19.1%) as intermediate risk. In contrast, of the 502 participants with MCI, 117 (23.3%) were identified as low risk, 180 (35.9%) as high risk, and 205 (40.8%) as intermediate risk.

### 3.3 Second model selection step in training cohort (ADNI)

**FIGURE 3:**
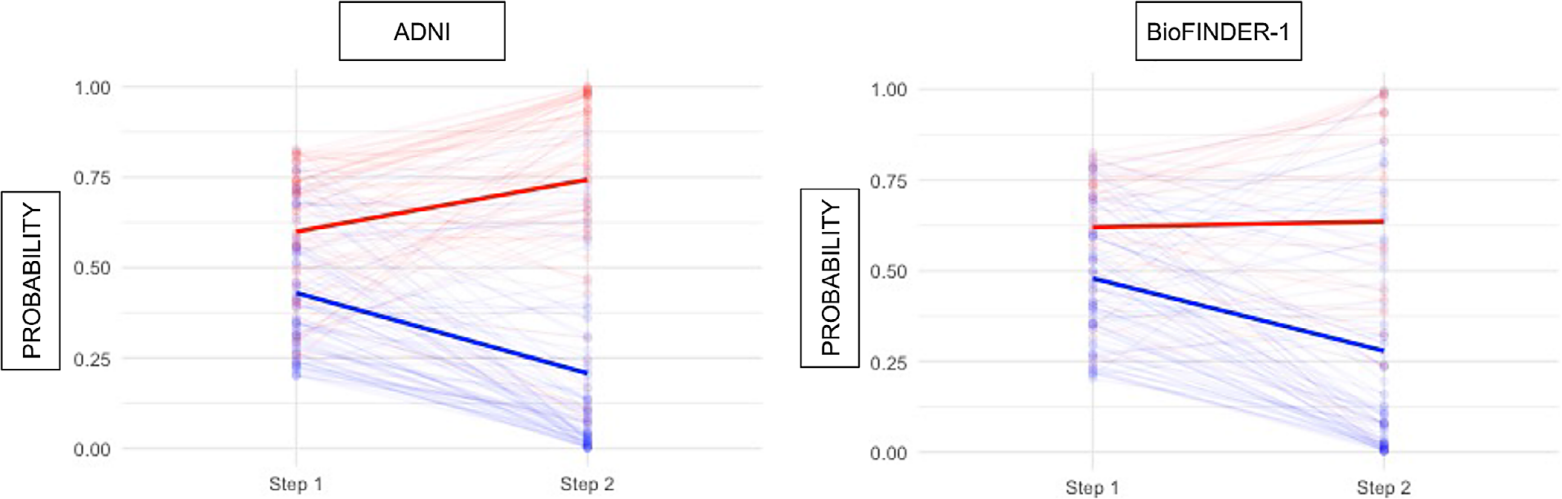
Probabilities of progressing to dementia within 4 years in Steps 1 and 2 for individuals with intermediate risk in ADNI (*N* = 170) and BioFINDER‐1 (*N* = 126). Blue = non‐progressors, red = progressors. The bold line shows mean probabilities for progression to dementia.

For the second step in ADNI (Figure 2B), representing a 1‐year follow‐up visit with new cognitive testing, we included the participants with intermediate risk/probability of progressing to dementia according to the model in the first step (ie, those between the two cutoffs) (Supplementary File 2B). Participants with intermediate risk were included if they had undergone cognitive testing at a 1‐year follow‐up visit and were excluded if they had progressed to dementia by that time. Out of the initial cohort, 226 individuals (36.9%) fell within the intermediate‐risk range. Of these, 40 participants were excluded due to progression to dementia at the 1‐year follow‐up, and 16 were excluded due to missing data (Figure 1A). This resulted in 170 remaining participants for the second step of the model selection. In the second step of the two‐step model, we identified the best model for predicting progression to dementia within the remaining 3 years (while dementia status at 4 years remained the outcome of the model). The model included the tests TMTB, MMSE, ADAS delayed recall, and ADAS immediate recall, along with the changes in these test results over the 1‐year follow‐up period. In the logistic regression analysis, all predictors were statistically significant apart from the difference/change in TMTB (*p* = .06). The model demonstrated an AUC of 0.92 (95% CI: 0.87 to 0.96), a PPV of 74.2%, a NPV of 90.9%, and an accuracy of 81.9% using a cutoff at 90% sensitivity in ADNI. Figure 3A illustrates the changes in the probability of progression to dementia from Step 1 to Step 2 for individuals with intermediate risk in ADNI. The figure shows how intermediate probabilities in Step 1 are largely refined into more accurate and distinct probabilities (either lower or higher) in Step 2, facilitating clearer differentiation between those likely to progress to dementia and those expected to remain stable. Additionally, we present mean baseline cognitive test scores for all included participants in ADNI, categorized by their risk levels (low, intermediate, or high) for future dementia in Table S3.

### 3.4 Two‐step model replicated in validation cohort (BioFINDER‐1)

We then applied the two‐step model to the BioFINDER‐1 cohort, using the estimates from the logistic regression model and the cutoffs established in ADNI. In the first step (Figure 2C), the model achieved an AUC of 0.86 (95% CI: 0.82 to 0.89). In this analysis, among 176 individuals with SCD, 100 were classified as low risk, nine as high risk, and 67 as intermediate risk. Of the 216 individuals with MCI, 18 were classified as low risk, 95 as high risk, and 113 as intermediate risk. Among those predicted to be at high risk of progression to dementia, 74 individuals did progress to dementia within 4 years, while 30 did not (PPV = 71.2%). Among those predicted to be at low risk, 115 did not progress to dementia, and only three did progress within 4 years (NPV = 97.5%).

A total of 170 individuals from BioFINDER‐1 fell in between the thresholds of 95% sensitivity and specificity and were included in the 1‐year follow‐up if they had not progressed to dementia within the first year (six individuals progressed within 1 year) and had available cognitive assessment data (38 individuals had missing data) (Figure 1B). To replicate the second step (Figure 2D), we included the 126 participants from BioFINDER‐1 who were classified as intermediate risk in the first step and had predictor data. In this replication, the model achieved an AUC of 0.79 (95% CI: 0.70 to 0.88). Among these participants, 31 individuals classified as high risk progressed to dementia within 4 years, while 33 were at high risk but did not progress (PPV 48.4%). Fifty‐seven participants classified as low risk did not progress within 4 years, while five individuals classified as low risk did progress within 4 years (NPV 91.9%). The total accuracy (proportion of correctly classified participants out of all participants) from both models in BioFINDER‐1 (Step 1 + Step 2) was 79.6%, with a NPV of 95.6%, a PPV of 62.5%, a sensitivity of 92.9%, and a specificity of 73.2% (Tables 2 and 3). Figure 3B shows the changes in the probability of progression to dementia from Step 1 to Step 2 for individuals at intermediate risk in BioFINDER‐1. We present the number of individuals screened as high risk for dementia within 4 years depending on follow‐up diagnosis in BioFINDER‐1 in Table S4. In a subanalysis, we observed a numerically slightly higher PPV but lower NPV when testing the model on individuals followed for 6 or 8 years, respectively, or who progressed before (Tables S5 and S6). We also conducted a subanalysis to compare our two‐step model with a one‐step model, setting the probability threshold at 0.5 for conversion to dementia. The one‐step model showed lower overall accuracy: 83.2% versus 89.0% in the two‐step model in ADNI and 74.5% versus 79.6% in BioFINDER‐1 (Table S7).

**TABLE 2:** Participants stratified by risk according to model and progression to dementia at each step in ADNI and BioFINDER‐1. Step 1 shows number of individuals progressing versus not progressing to dementia within 4 years in groups at >95% sensitivity and >95% specificity level in ADNI and BioFINDER‐1 in Step 1. Step 2 shows number of individuals progressing versus not progressing to dementia within 3 years in the groups at the >90% sensitivity level in ADNI and BioFINDER‐1 in Step 2. Combined steps show number of individuals progressing versus not progressing to dementia within 4 years in the groups at the >95% sensitivity and >95% specificity level in ADNI and BioFINDER‐1 in Step 1 combined with the >90% sensitivity level within 3 years in Step 2.

	Study							
	ADNI				BioFINDER‐1			
	Progress within 4 years	Progress within 1 year	Not progressing	Total progressors + non‐progressors	Progress within 4 years	Progress within 1 year	Not progressing	Total progressors + non‐progressors
**Step 1**								
High risk	167	77	15	182	74	9	30	104
Low risk	15	5	189	204	3	0	115	118
Total (high or low risk)	182	82	204	386	77	9	145	222
Intermediate risk	119	40	107	226	64	6	106	170
**Step 2** ^a^								
High risk	69	–	24	93	31	–	33	64
Low risk	7	–	70	77	5	–	57	62
Total	76	–	94	170	36	–	90	126
**Combined steps**								
High risk	236	–	39	275	105	–	63	168
Low risk	22	–	259	281	8	–	172	180
Total	258	–	298	556	113	–	235	348

^a^ *Note*: that number of participants at Step 2 differs from the intermediate group at Step 1 due to missingness in follow‐up test data.

**TABLE 3:** Step 1 shows levels calculated from individuals progressing versus not progressing to dementia within 4 years in groups at >95% sensitivity and >95% specificity level in ADNI and BioFINDER‐1 in Step 1. Step 2 shows levels calculated from individuals progressing versus not progressing to dementia within 3 years in groups at >90% sensitivity level in ADNI and BioFINDER‐1 in Step 2. Combined steps show levels calculated from individuals progressing versus not progressing to dementia within 4 years in groups at >95% sensitivity and >95% specificity level in ADNI and BioFINDER‐1 in Step 1 combined with >90% sensitivity level within 3 years in Step 2.

	Step 1		Step 2		Combined steps	
	ADNI	BF‐1	ADNI	BF‐1	ADNI	BF‐1
Prevalence of dementia progression	47.2%	34.7%	44.7%	28.6%	46.4%	32.5%
Sensitivity	91.8%	96.1%	90.8%	86.1%	91.5%	92.9%
Specificity	92.6%	79.3%	74.5%	63.3%	86.9%	73.2%
PPV	91.8%	71.2%	74.2%	48.4%	85.8%	62.5%
NPV	92.6%	97.5%	90.9%	91.9%	92.2%	95.6%
Accuracy	92.2%	85.1%	81.8%	69.8%	89.0%	79.6%

Abbreviations: ADNI, Alzheimer's Disease Neuroimaging Initiative; BF‐1, BioFINDER‐1; NPV: negative predictive value; PPV, positive predictive value.

### 3.5 Supplementary model selection using MRI

As a supplementary analysis, we compared the two‐step model with a one‐step model that included MRI data (cortical thickness in a temporal composite region)^37^ (Supplementary File 2C, Tables S8–S9, and Figure S1). The best model included the temporal composite as well as test results of ADAS immediate recall, ADAS delayed recall, Animal Fluency, MMSE, and TMTB. The overall accuracy of the one‐step model for ADNI was 85.3%, with a PPV of 80.5% and a NPV of 90.5%. For BioFINDER‐1, the model had an accuracy of 65.0%, a PPV of 50.0%, and a NPV of 97.4%.

### 3.6 Using the two‐step model for individualized prediction of future dementia

To provide an individualized risk of progressing to dementia using this two‐step approach, we developed an app available for research and educational purposes at https://brainapps.shinyapps.io/PredictAllCauseDementia. This app aids in predicting whether individuals are at low, intermediate, or high risk of progressing to dementia. To use the application, simply enter the individual's cognitive test results from the ADAS delayed recall, ADAS immediate recall, TMTB, MMSE, and Animal Fluency. The application then calculates the probability of the individual progressing to dementia within 4 years. Additionally, there is a tab for entering 1‐year follow‐up scores for the first four tests, based on the logistic regression model. The app uses logistic regression models to fit with the ADNI data described in the paper. Predicted probabilities for new data (defined by the user) are calculated using the coefficients from the logistic regression models.

## 4 DISCUSSION

We have developed a novel two‐step model designed to aid in the prognostic evaluation of patients presenting with cognitive symptoms, preferably to be used in primary care settings (Figure 2A–D and Supplementary Files 2A–B). The total two‐step model had a high NPV for individuals not progressing to dementia within 4 years, meaning the model effectively identified individuals who were unlikely to progress to dementia (92.2% in ADNI and 95.6% when replicated in BioFINDER‐1). The PPV was moderate, meaning it showed a reasonable ability to identify individuals who will progress to dementia within 4 years (85.8% in ADNI and 62.5% when replicated in BioFINDER‐1). Patients identified as being at high risk of dementia using this two‐step model should thus be further evaluated, for example, in specialized care, to identify those with a prodromal dementia.

Already at the first step (initial test visit), a high NPV was achieved, which is particularly relevant in clinical practice for reassuring patients they are at low risk of developing dementia. This is especially important in primary care (>76% of participants in the validation cohort were referred directly to primary care). The overall lower PPV indicates that this workflow alone is not sufficient for identifying those with underlying prodromal dementia. Instead, it can be used to identify individuals at increased risk who would likely benefit from a more thorough secondary care work‐up involving advanced biomarker investigations, which typically exhibit high PPVs, thereby enhancing overall diagnostic accuracy.

Those at intermediate risk in this workflow are recommended to undergo a second cognitive test visit after 1 year. The second step of the model incorporated changes in cognitive testing into the best predictive model, aligning with previous research showing that the prediction of progression to dementia is improved if longitudinal information is incorporated.^24^, ^25^, ^26^ Regarding the composition of cognitive stages at the second step, there were few individuals with SCD included in ADNI (five individuals), as many SCD individuals were screened as low risk in the first step (*N* = 87 out of 103), while two were screened as high risk. Although the second step increased the overall accuracy, it came with the drawback that some individuals had already progressed to dementia when it was time for the 1‐year follow‐up testing needed for Step 2. In an era where new disease‐modifying treatment is being implemented, it is important to identify patients as early as possible to achieve the best treatment effect. In countries or regions where secondary care allows for a higher number of referrals, it could thus be an option for primary care physicians to refer patients at either high or intermediate risk already after Step 1.

To our knowledge, no previous studies utilized two‐step cognitive test models to assess the individual risk of developing future dementia. Previous studies investigated which predictors were most important for assessing the risk of progression to dementia, often showing that a combination of memory tests, executive tests, and an instrumental activities of daily living (IADL) scale are significant factors.^42^ Other studies highlighted the importance of the MMSE, verbal fluency tests, and an IADL scale.^43^ However, these studies did not present models that, following adequate validation, could be used in clinical practice to discriminate between individuals with low versus high risk of progression to dementia.

One study investigated a model for the prediction of AD dementia using scores of global cognitive function (MMSE and Cambridge Cognitive Examination‐Revised), a test of verbal episodic memory and psychomotor speed (TMTA), including changes in test scores over a 1‐year follow‐up.^44^ While our study included some similar tests covering global cognitive assessment, verbal episodic memory, and psychomotor speed (MMSE, ADAS delayed recall, and TMTB), our battery additionally included markers of verbal fluency (Animal Fluency) and learning memory (ADAS immediate recall). Additionally, our study used TMTB, which assesses cognitive flexibility and executive function, offering more information compared to TMTA.^45^ The broader, data‐driven selection of tests in our study might reflect the differences in outcomes, enabling the prediction of progression to non‐AD neurodegenerative diseases as well (Table S4).

Cognitive decline in dementia affects patients differently depending on the underlying pathology. In AD, patients typically present with episodic memory complaints, which progress to difficulties in speech production, orientation, calculation, and learning disabilities over time.^46^, ^47^ Previous studies identified TMTB, MMSE, and ADAS delayed recall as accurate for predicting AD dementia.^8^, ^39^ For vascular cognitive impairment, individuals often experience declines in executive function, speed, or attention,^48^ with TMTB frequently affected.^49^, ^50^ DLB often impacts attention, executive function, language, behavior, visuospatial function, and memory.^51^ In Lewy body disease, the ADAS delayed recall test is affected in already asymptomatic stages^52^ as well as in MCI and dementia,^53^ and TMTB is clearly affected in DLB.^54^ Patients with behavioral variant FTD (bvFTD) typically show declines in executive function while generally retaining memory and visuospatial function.^55^ In prodromal FTD, TMTB shows early changes.^56^

Previous studies assessed individualized risk of all‐cause dementia using CSF biomarkers, hippocampal volume (visualized on MRI scans), and cognitive assessments using the application: https://www.adappt.health/.^57^ However, this method is not suitable for triaging in primary care where CSF biomarkers are not available. MRI is, however, sometimes used in initial work‐ups. We therefore performed a supplemental analysis including MRI in a one‐step model for comparison with our two‐step cognitive test model. When incorporating temporal cortical thickness in the model and replicating in BioFINDER‐1, the PPV was low (50.0%), and overall accuracy was 65.0%, indicating that MRI might not contribute to predicting all‐cause dementia (Tables S8 and S9).

An advantage of our method is that it is a non‐invasive and inexpensive triaging method. The selected cognitive tests in the model are relatively few (five brief cognitive tests) and are easily implemented in a primary care setting, as was done, for example, in the BioFINDER‐Primary Care study (NCT06120361). Such an algorithm could be valuable in clinical scenarios where an increasing population seeks medical help for cognitive symptoms and wishes to conduct blood tests to examine for AD and receive anti‐amyloid therapies. The study included participants with subjective and objective cognitive impairment and was replicated in a group of individuals seeking healthcare for actual subjective or objective cognitive impairment, similar to the population intended for the method. Future studies should investigate whether our model could aid in identifying which patients should be tested with blood markers, potentially improving the overall PPV regarding progression to dementia.

In a subanalysis, we investigated the performance of the two‐step model in BioFINDER‐1, including participants followed for either 6 or 8 years or who progressed before these time points. Due to the small number of non‐progressors in the later years (182 for the 6‐year follow‐up and 86 for the 8‐year follow‐up), the results should be interpreted with caution. However, the PPV increased numerically from 62.2% in the 4‐year prediction to 77.3% in the 6‐year prediction and 94.1% for the 8‐year follow‐up. Conversely, the NPV decreased from 95.6% in the 4‐year prediction to 83.8% in the 6‐year prediction and 67.3% in the 8‐year prediction. These results indicate that individuals at high risk are highly likely to progress to dementia, if not within 4 years, then at least within 6 to 8 years. Conversely, low‐risk individuals can be reassured for the next 4 years, but they should be advised to seek care again if symptoms progress.

A limitation of the study is the rather small sample sizes from ADNI and BioFINDER‐1 during the second step of the model (*N* = 126 to 170), predicting dementia for individuals at intermediate risk at baseline. This limitation is mainly caused by missing data from cognitive tests at follow‐up. Although a lower number of individuals at this stage is expected and desired for clinical practice, the reduced sample size is a constraint. Despite these smaller sample sizes, our results were well replicated in the validation cohort, which minimized the risk of chance findings.

Another limitation of the study is the limited number of cognitive tests available for inclusion in the model selection from both ADNI and BioFINDER‐1. Although the selected cognitive tests are well established and validated, other cognitive tests could have performed equally or even better. Additionally, the results for TMTB at 1‐year follow‐up were calculated from the difference in baseline and 2‐year follow‐up scores, divided by 2 to estimate the mean annual change. This approach may not accurately reflect the actual 1‐year test difference, as cognitive decline might occur in a stepwise fashion rather than a linear progression. However, as we used ADNI as the training study and BioFINDER‐1 to replicate the findings, this would not affect the prediction model but could influence the AUC when replicating the model in BioFINDER‐1.

The ADNI and BioFINDER‐1 cohorts differ in their average years of education, with the ADNI cohort having a mean of 16.3 years (SD 2.7) and the BioFINDER‐1 11.8 years (SD 3.5) (Table S1). This educational difference could have influenced the results, potentially leading to better test scores in ADNI. However, demographic factors, including education level, were included in the calculations, but they were not selected in the MuMIn model selection for the best dementia prediction model. Future studies should evaluate the reproducibility of the model in more diverse cohorts with varying education levels.

To conclude, logistic regression models were developed and validated using brief cognitive tests to predict progression to dementia, categorizing individuals into low‐, intermediate‐, and high‐risk groups. We also created an app to facilitate the entry of test results and predict future dementia progression. Future studies should examine these findings in larger, more diverse cohorts to further validate and refine the model.

## Collaborators

Michael Weiner, MD (University of California [UC] San Francisco, Principal Investigator, Executive Committee); Paul Aisen, MD (UC San Diego, ADCS PI and Director of Coordinating Center Clinical Core, Executive Committee, Clinical Core Leaders); Ronald Petersen, MD, PhD (Mayo Clinic, Rochester, Executive Committee, Clinical Core Leader); Clifford R. Jack, Jr., MD (Mayo Clinic, Rochester, Executive Committee, MRI Core Leader); William Jagust, MD (UC Berkeley, Executive Committee; PET Core Leader); John Q. Trojanowki, MD, PhD (University of Pennsylvania, Executive Committee, Biomarkers Core Leader); Arthur W. Toga, PhD (University of Southern California [USC], Executive Committee, Informatics Core Leader); Laurel Beckett, PhD (UC Davis, Executive Committee, Biostatistics Core Leader); Robert C. Green, MD, MPH (Brigham and Women's Hospital, Harvard Medical School, Executive Committee and Chair of Data and Publication Committee); Andrew J. Saykin, PsyD (Indiana University, Executive Committee, Genetics Core Leader); John Morris, MD (Washington University St. Louis, Executive Committee, Neuropathology Core Leader); Leslie M. Shaw (University of Pennsylvania, Executive Committee, Biomarkers Core Leader); Enchi Liu, PhD (Janssen Alzheimer Immunotherapy, ADNI 2 Private Partner Scientific Board Chair); Tom Montine, MD, PhD (University of Washington); Ronald G. Thomas, PhD (UC San Diego); Michael Donohue, PhD (UC San Diego); Sarah Walter, MSc (UC San Diego); Devon Gessert (UC San Diego); Tamie Sather, MS (UC San Diego); Gus Jiminez, MBS (UC San Diego); Danielle Harvey, PhD (UC Davis); Michael Donohue, PhD (UC San Diego); Matthew Bernstein, PhD (Mayo Clinic, Rochester); Nick Fox, MD (University of London); Paul Thompson, PhD (USC School of Medicine); Norbert Schuff, PhD (UCSF MRI); Charles DeCArli, MD (UC Davis); Bret Borowski, RT (Mayo Clinic); Jeff Gunter, PhD (Mayo Clinic); Matt Senjem, MS (Mayo Clinic); Prashanthi Vemuri, PhD (Mayo Clinic); David Jones, MD (Mayo Clinic); Kejal Kantarci (Mayo Clinic); Chad Ward (Mayo Clinic); Robert A. Koeppe, PhD (University of Michigan, PET Core Leader); Norm Foster, MD (University of Utah); Eric M. Reiman, MD (Banner Alzheimer's Institute); Kewei Chen, PhD (Banner Alzheimer's Institute); Chet Mathis, MD (University of Pittsburgh); Susan Landau, PhD (UC Berkeley); Nigel J. Cairns, PhD, MRCPath (Washington University St. Louis); Erin Householder (Washington University St. Louis); Lisa Taylor Reinwald, BA, HTL (Washington University St. Louis); Virginia Lee, PhD, MBA (UPenn School of Medicine); Magdalena Korecka, PhD (UPenn School of Medicine); Michal Figurski, PhD (University of Pennsylvania School of Medicine); Karen Crawford (USC); Scott Neu, PhD (USC); Tatiana M. Foroud, PhD (Indiana University); Steven Potkin, MD UC (UC Irvine); Li Shen, PhD (Indiana University); Faber Kelley, MS, CCRC (Indiana University); Sungeun Kim, PhD (Indiana University); Kwangsik Nho, PhD (Indiana University); Zaven Kachaturian, PhD (Khachaturian, Radebaugh & Associates, Inc. and Alzheimer's Association's Ronald and Nancy Reagan Research Institute); Richard Frank, MD, PhD (General Electric); Peter J. Snyder, PhD (Brown University); Susan Molchan, PhD (National Institute on Aging/National Institutes of Health); Jeffrey Kaye, MD (Oregon Health and Science University); Joseph Quinn, MD (Oregon Health and Science University); Betty Lind, BS (Oregon Health and Science University); Raina Carter, BA (Oregon Health and Science University); Sara Dolen, BS (Oregon Health and Science University); Lon S. Schneider, MD (USC); Sonia Pawluczyk, MD (USC); Mauricio Beccera, BS (USC); Liberty Teodoro, RN (USC); Bryan M. Spann, DO, PhD (USC); James Brewer, MD, PhD (UC San Diego); Helen Vanderswag, RN (UC San Diego); Adam Fleisher, MD (UC San Diego); Judith L. Heidebrink, MD, MS (University of Michigan); Joanne L. Lord, LPN, BA, CCRC (University of Michigan); Ronald Petersen, MD, PhD (Mayo Clinic, Rochester); Sara S. Mason, RN (Mayo Clinic, Rochester); Colleen S. Albers, RN (Mayo Clinic, Rochester); David Knopman, MD (Mayo Clinic, Rochester); Kris Johnson, RN (Mayo Clinic, Rochester); Rachelle S. Doody, MD, PhD (Baylor College of Medicine); Javier Villanueva Meyer, MD (Baylor College of Medicine); Munir Chowdhury, MBBS, MS (Baylor College of Medicine); Susan Rountree, MD (Baylor College of Medicine); Mimi Dang, MD (Baylor College of Medicine); Yaakov Stern, PhD (Columbia University Medical Center); Lawrence S. Honig, MD, PhD (Columbia University Medical Center); Karen L. Bell, MD (Columbia University Medical Center); Beau Ances, MD (Washington University, St. Louis); John C. Morris, MD (Washington University, St. Louis); Maria Carroll, RN, MSN (Washington University, St. Louis); Sue Leon, RN, MSN (Washington University, St. Louis); Erin Householder, MS, CCRP (Washington University, St. Louis); Mark A. Mintun, MD (Washington University, St. Louis); Stacy Schneider, APRN, BC, GNP (Washington University, St. Louis); Angela Oliver, RN, BSN, MSG; Daniel Marson, JD, PhD (University of Alabama Birmingham); Randall Griffith, PhD, ABPP (University of Alabama Birmingham); David Clark, MD (University of Alabama Birmingham); David Geldmacher, MD (University of Alabama Birmingham); John Brockington, MD (University of Alabama Birmingham); Erik Roberson, MD (University of Alabama Birmingham); Hillel Grossman, MD (Mount Sinai School of Medicine); Effie Mitsis, PhD (Mount Sinai School of Medicine); Leyla deToledo‐Morrell, PhD (Rush University Medical Center); Raj C. Shah, MD (Rush University Medical Center); Ranjan Duara, MD (Wien Center); Daniel Varon, MD (Wien Center); Maria T. Greig, HP (Wien Center); Peggy Roberts, CNA (Wien Center); Marilyn Albert, PhD (Johns Hopkins University); Chiadi Onyike, MD (Johns Hopkins University); Daniel D'Agostino II, BS (Johns Hopkins University); Stephanie Kielb, BS (Johns Hopkins University); James E. Galvin, MD, MPH (New York University); Dana M. Pogorelec (New York University); Brittany Cerbone (New York University); Christina A. Michel (New York University); Henry Rusinek, PhD (New York University); Mony J de Leon, EdD (New York University); Lidia Glodzik, MD, PhD (New York University); Susan De Santi, PhD (New York University); P. Murali Doraiswamy, MD (Duke University Medical Center); Jeffrey R. Petrella, MD (Duke University Medical Center); Terence Z. Wong, MD (Duke University Medical Center); Steven E. Arnold, MD (University of Pennsylvania); Jason H. Karlawish, MD (University of Pennsylvania); David Wolk, MD (University of Pennsylvania); Charles D. Smith, MD (University of Kentucky); Greg Jicha, MD (University of Kentucky); Peter Hardy, PhD (University of Kentucky); Partha Sinha, PhD (University of Kentucky); Elizabeth Oates, MD (University of Kentucky); Gary Conrad, MD (University of Kentucky); Oscar L. Lopez, MD (University of Pittsburgh); MaryAnn Oakley, MA (University of Pittsburgh); Donna M. Simpson, CRNP, MPH (University of Pittsburgh); Anton P. Porsteinsson, MD (University of Rochester Medical Center); Bonnie S. Goldstein, MS, NP (University of Rochester Medical Center); Kim Martin, RN (University of Rochester Medical Center); Kelly M. Makino, BS (University of Rochester Medical Center); M. Saleem Ismail, MD (University of Rochester Medical Center); Connie Brand, RN (University of Rochester Medical Center); Ruth A. Mulnard, DNSc, RN, FAAN (UC Irvine); Gaby Thai, MD (UC Irvine); Catherine Mc Adams Ortiz, MSN, RN, A/GNP (UC Irvine); Kyle Womack, MD (University of Texas Southwestern Medical School); Dana Mathews, MD, PhD (University of Texas Southwestern Medical School); Mary Quiceno, MD (University of Texas Southwestern Medical School); Ramon Diaz Arrastia, MD, PhD (University of Texas Southwestern Medical School); Richard King, MD (University of Texas Southwestern Medical School); Myron Weiner, MD (University of Texas Southwestern Medical School); Kristen Martin Cook, MA (University of Texas Southwestern Medical School); Michael DeVous, PhD (University of Texas Southwestern Medical School); Allan I. Levey, MD, PhD (Emory University); James J. Lah, MD, PhD (Emory University); Janet S. Cellar, DNP, PMHCNS BC (Emory University); Jeffrey M. Burns, MD (University of Kansas, Medical Center); Heather S. Anderson, MD (University of Kansas, Medical Center); Russell H. Swerdlow, MD (University of Kansas, Medical Center); Liana Apostolova, MD (UC Los Angeles [UCLA]); Kathleen Tingus, PhD (UCLA); Ellen Woo, PhD (UCLA); Daniel H.S. Silverman, MD, PhD (UCLA); Po H. Lu, PsyD (UCLA); George Bartzokis, MD (UCLA); Neill R. Graff Radford, MBBCH, FRCP (London) (Mayo Clinic, Jackson2 Groups Acknowledgements Journal Formatville); Francine Parfitt, MSH, CCRC (Mayo Clinic, Jacksonville); Tracy Kendall, BA, CCRP (Mayo Clinic, Jacksonville); Heather Johnson, MLS, CCRP (Mayo Clinic, Jacksonville); Martin R. Farlow, MD (Indiana University); Ann Marie Hake, MD (Indiana University); Brandy R. Matthews, MD (Indiana University); Scott Herring, RN, CCRC (Indiana University); Cynthia Hunt, BS, CCRP (Indiana University); Christopher H. van Dyck, MD (Yale University School of Medicine); Richard E. Carson, PhD (Yale University School of Medicine); Martha G. MacAvoy, PhD (Yale University School of Medicine); Howard Chertkow, MD (McGill University, Montreal Jewish General Hospital); Howard Bergman, MD (McGill University, Montreal Jewish General Hospital); Chris Hosein, Med (McGill University, Montreal Jewish General Hospital); Sandra Black, MD, FRCPC (Sunnybrook Health Sciences, Ontario); Dr. Bojana Stefanovic (Sunnybrook Health Sciences, Ontario); Curtis Caldwell, PhD (Sunnybrook Health Sciences, Ontario); Ging Yuek Robin Hsiung, MD, MHSc, FRCPC (University of British Columbia [UBC] Clinic for Alzheimer Disease and Related Disorders); Howard Feldman, MD, FRCPC (UBC Clinic for Alzheimer Disease and Related Disorders); Benita Mudge, BS (UBC Clinic for Alzheimer Disease and Related Disorders); Michele Assaly, MA Past (UBC Clinic for Alzheimer Disease and Related Disorders); Andrew Kertesz, MD (Cognitive Neurology St. Joseph's, Ontario); John Rogers, MD (Cognitive Neurology St. Joseph's, Ontario); Dick Trost, PhD (Cognitive Neurology St. Joseph's, Ontario); Charles Bernick, MD (Cleveland Clinic Lou Ruvo Center for Brain Health); Donna Munic, PhD (Cleveland Clinic Lou Ruvo Center for Brain Health); Diana Kerwin, MD (Northwestern University); Marek Marsel Mesulam, MD (Northwestern University); Kristine Lipowski, BA (Northwestern University); Chuang Kuo Wu, MD, PhD (Northwestern University); Nancy Johnson, PhD (Northwestern University); Carl Sadowsky, MD (Premiere Research Institute [Palm Beach Neurology]); Walter Martinez, MD (Premiere Research Institute [Palm Beach Neurology]); Teresa Villena, MD (Premiere Research Institute [Palm Beach Neurology]); Raymond Scott Turner, MD, PhD (Georgetown University Medical Center); Kathleen Johnson, NP (Georgetown University Medical Center); Brigid Reynolds, NP (Georgetown University Medical Center); Reisa A. Sperling, MD (Brigham and Women's Hospital); Keith A. Johnson, MD (Brigham and Women's Hospital); Gad Marshall, MD (Brigham and Women's Hospital); Meghan Frey (Brigham and Women's Hospital); Jerome Yesavage, MD (Stanford University); Joy L. Taylor, PhD (Stanford University); Barton Lane, MD (Stanford University); Allyson Rosen, PhD (Stanford University); Jared Tinklenberg, MD (Stanford University); Marwan N. Sabbagh, MD (Banner Sun Health Research Institute); Christine M. Belden, PsyD (Banner Sun Health Research Institute); Sandra A. Jacobson, MD (Banner Sun Health Research Institute); Sherye A. Sirrel, MS (Banner Sun Health Research Institute); Neil Kowall, MD (Boston University); Ronald Killiany, PhD (Boston University); Andrew E. Budson, MD (Boston University); Alexander Norbash, MD (Boston University); Patricia Lynn Johnson, BA (Boston University); Thomas O. Obisesan, MD, MPH (Howard University); Saba Wolday, MSc (Howard University); Joanne Allard, PhD (Howard University); Alan Lerner, MD (Case Western Reserve University); Paula Ogrocki, PhD (Case Western Reserve University); Leon Hudson, MPH (Case Western Reserve University); Evan Fletcher, PhD (UC Davis Sacramento); Owen Carmichael, PhD (UC Davis Sacramento); John Olichney, MD (UC Davis Sacramento); Charles DeCarli, MD (UC Davis Sacramento); Smita Kittur, MD (Neurological Care of CNY); Michael Borrie, MB ChB (Parkwood Hospital); T.Y. Lee, PhD (Parkwood Hospital); Dr. Rob Bartha, PhD (Parkwood Hospital); Sterling Johnson, PhD (University of Wisconsin); Sanjay Asthana, MD (University of Wisconsin); Cynthia M. Carlsson, MD (University of Wisconsin); Steven G. Potkin, MD (UC Irvine BIC); Adrian Preda, MD (UC Irvine BIC); Dana Nguyen, PhD (UC Irvine BIC); Pierre Tariot, MD (Banner Alzheimer's Institute); Adam Fleisher, MD (Banner Alzheimer's Institute); Stephanie Reeder, BA (Banner Alzheimer's Institute); Vernice Bates, MD (Dent Neurologic Institute); Horacio Capote, MD (Dent Neurologic Institute); Michelle Rainka, PharmD, CCRP (Dent Neurologic Institute); Douglas W. Scharre, MD (The Ohio State University); Maria Kataki, MD, PhD (The Ohio State University); Anahita Adeli, MD (The Ohio State University); Earl A. Zimmerman, MD (Albany Medical College); Dzintra Celmins, MD (Albany Medical College); Alice D. Brown, FNP (Albany Medical College); Godfrey D. Pearlson, MD (Hartford Hospital, Olin Neuropsychiatry Research Center); Karen Blank, MD (Hartford Hospital, Olin Neuropsychiatry Research Center); Karen Anderson, RN (Hartford Hospital, Olin Neuropsychiatry Research Center); Robert B. Santulli, MD (Dartmouth Hitchcock Medical Center); Tamar J. Kitzmiller (Dartmouth Hitchcock Medical Center); Eben S. Schwartz, PhD (Dartmouth Hitchcock Medical Center); Kaycee M. Sink, MD, MAS (Wake Forest University Health Sciences); Jeff D. Williamson, MD, MHS (Wake Forest University Health Sciences); Pradeep Garg, PhD (Wake Forest University Health Sciences); Franklin Watkins, MD (Wake Forest University Health Sciences); Brian R. Ott, MD (Rhode Island Hospital); Henry Querfurth, MD (Rhode Island Hospital); Geoffrey Tremont, PhD (Rhode Island 3 Groups Acknowledgements Journal FormatHospital); Stephen Salloway, MD, MS (Butler Hospital); Paul Malloy, PhD (Butler Hospital); Stephen Correia, PhD (Butler Hospital); Howard J. Rosen, MD (UC San Francisco); Bruce L. Miller, MD (UC San Francisco); Jacobo Mintzer, MD, MBA (Medical University South Carolina); Kenneth Spicer, MD, PhD (Medical University South Carolina); David Bachman, MD (Medical University South Carolina); Elizabether Finger, MD (St. Joseph's Health Care); Stephen Pasternak, MD (St. Joseph's Health Care); Irina Rachinsky, MD (St. Joseph's Health Care); John Rogers, MD (St. Joseph's Health Care); Andrew Kertesz, MD (St. Joseph's Health Care); Dick Drost, MD (St. Joseph's Health Care); Nunzio Pomara, MD (Nathan Kline Institute); Raymundo Hernando, MD (Nathan Kline Institute); Antero Sarrael, MD (Nathan Kline Institute); Susan K. Schultz, MD (University of Iowa College of Medicine, Iowa City); Laura L. Boles Ponto, PhD (University of Iowa College of Medicine, Iowa City); Hyungsub Shim, MD (University of Iowa College of Medicine, Iowa City); Karen Elizabeth Smith, RN (University of Iowa College of Medicine, Iowa City); Norman Relkin, MD, PhD (Cornell University); Gloria Chaing, MD (Cornell University); Lisa Raudin, PhD (Cornell University); Amanda Smith, MD (University of South Florida [USF] Health Byrd Alzheimer's Center and Research Institute); Kristin Fargher, MD (USF Health Byrd Alzheimer's Center and Research Institute); Balebail Ashok Raj, MD (USF Health Byrd Alzheimer's Center and Research Institute).

## CONFLICT OF INTEREST STATEMENT

Emma Borland, Erik Stomrud, and Pontus Tideman have no conflicts of interest to disclose. Niklas Mattson‐Carlgren has a consulting agreement with Biogen. Oskar Hansson has acquired research support (for the institution) from ADx, AVID Radiopharmaceuticals, Biogen, Eli Lilly, Eisai, Fujirebio, GE Healthcare, Pfizer, and Roche. In the past 2 years, he has received consultancy/speaker fees from AC Immune, Alzpath, BioArctic, Biogen, Bristol Meyer Squibb, Cerveau, Eisai, Eli Lilly, Fujirebio, Merck, Novartis, Novo Nordisk, Roche, Sanofi, and Siemens. Sebastian Palmqvist receives research support (for the institution) from Avid and ki elements/ADDF. In the past 2 years, he has received consultancy/speaker fees from Bioartic, Biogen, Eisai, Eli Lilly, and Roche. Author disclosures are available in the Supporting information.

## CONSENT STATEMENT

We utilized OpenAI's ChatGPT to improve language and clarity of the manuscript writing.

## Supporting information



Supporting Information

Supporting Information

Supporting Information

Supporting Information
