# Supplementary material for: Individualized, cross‐validated prediction of future dementia using cognitive assessments in people with mild cognitive symptoms
Source: Alzheimers Dement. 2024 Oct 17;20(12):8625–38. doi: 10.1002/alz.14305 (PMC11667515; doi:10.1002/alz.14305)
Supplement: Supplementary file 1 — Supporting Information [file ALZ-20-8625-s001.docx]

**Supplementary File 1**

**The inclusion criteria for both the SCD and MCI cohorts in ADNI were:**

1. Geriatric Depression Scale less than 6
2. Age between 55-90 for MCI and 65-90 for SCD
3. Study partner is available
4. Visual and auditory acuity adequate for neuropsychological testing
5. Good general health with no diseases expected to interfere with the study
6. Willing and able to participate in a longitudinal imaging study
7. Hachinski less than or equal to 4
8. Completed six grades of education or has a good work history (sufficient to exclude mental retardation)
9. Must speak English or Spanish fluently
10. Agrees to collection of blood for genetic analyses and biomarker testing
11. Agrees to at least one lumbar puncture for the collection of CSF

**The classification of SCD was introduced during the recruitment process of ADNI-2. The inclusion criteria for SCD were:**

1. "Self-referrals" that have a significant subjective memory concern
2. Significant memory concern confirmed by a Cognitive Change Index score of ≥16
3. Normal memory function score on Wechsler Memory Scale (adjusted for education)
4. MMSE score between 24 and 30 (inclusive)
5. Clinical Dementia Rating (CDR) = 0
6. Cognitively normal, based on the absence of significant memory impairment in cognitive function or activities of daily living
7. Stability of permitted medications for 4 weeks

**The inclusion criteria for MCI were:**

1. Participant must have a subjective memory concern as reported by participant, study partner, or clinician
2. Abnormal memory function score on Wechsler Memory Scale (adjusted for education)
3. MMSE score between 24 and 30 (inclusive)
4. CDR = 0.5 (Memory Box score must be at least 0.5)
5. General cognition and functional performance sufficiently preserved such that a diagnosis of AD cannot be made by the site physician at the time of the screening visit
6. Stability of Permitted Medications for 4 weeks.

**Exclusion criteria for both SCD and MCI were:**

1. Screening/baseline MRI scan with evidence of infection, infarction, or other focal lesions
2. Contraindications for MRI
3. Major depression or bipolar disorder within the past 1 year
4. Currently treated with medication for obsessive-compulsive disorder or attention deficit disorder
5. History of schizophrenia
6. History of alcohol or substance abuse or dependence within the past 2 years
7. Any significant systemic illness or unstable medical condition which could lead to difficulty complying with the protocol
8. Clinically significant abnormalities in vitamin B12, or TFTs that might interfere with the study
9. Current use of specific psychoactive medications (e.g., certain antidepressants, neuroleptics, chronic anxiolytics or sedative hypnotics, etc.)
10. Current use of warfarin or similar anticoagulants (exclusionary for lumbar puncture)
11. Any significant neurologic disease, such as Parkinson's disease, multi-infarct dementia, Huntington's disease, normal pressure hydrocephalus, brain tumor, progressive supranuclear palsy, seizure disorder, subdural hematoma, multiple sclerosis, or history of significant head trauma followed by persistent neurologic defaults or known structural brain abnormalities.

**Study subjects BioFINDER-1**

**The inclusion criteria for BioFINDER-1 subjects with mild cognitive symptoms were**

1. Referred to the memory clinic at Skåne University Hospital or Ängelholm hospital in Sweden due to cognitive symptoms experienced by the patient and/or informant. These symptoms did not have to be memory complaints, but could also be executive, visuo-spatial, language, praxis or psychomotor complaints
2. Age between 60 and 80 years
3. MMSE score of 24 – 30 points at baseline visit
4. Do not fulfill the criteria for any dementia
5. Speaks and understands Swedish to the extent that an interpreter not was necessary for the patient to fully understand the study information and neuropsychological tests

**Exclusion criteria for BioFINDER-1 subjects with mild cognitive symptoms were**

1. Significant unstable systemic illness or organ failure, such as terminal cancer, that makes it difficult to participate in the study
2. Current significant alcohol or substance misuse
3. Refusing lumbar puncture or neuropsychological assessment
4. The cognitive impairment at baseline visit can with certainty be explained by another condition or disease such as normal pressure hydrocephalus, major cerebral hemorrhage, brain infection, brain tumor, multiple sclerosis, epilepsy, psychotic disorders, severe depression, alcohol abuse the last five years, on-going medication with drugs that invariably cause cognitive impairment (such as high-dose benzodiazepines)

**Supplementary File 2**

Supplement 2A*. Step 1. Best model for predicting progression to dementia within four years from a baseline visit.*

**Best model fit in ADNI:**

3.86 + 0.408*ADAS immediate + 0.0117*TMT B – 0.0747*Animal Fluency – 0.263*MMSE + 0.315*ADAS delayed

**Best model fit in ADNI:**

5.06 + ADAS immediate*0.800 + ADAS immediate diff*0.507 +

TMT B*0.0150 + TMT B diff*0.0117 –

MMSE*0.517 – MMSE diff*0.542

+ ADAS delayed*0.563 + ADAS delayed diff*0.728

Supplement 2B*. Step 2. Best model for predicting a progression to dementia within three years after baseline and a 1-year follow-up visit.*

**Best model fit in ADNI:**

18.7 + 0.00918*TMT B + 0.271*Adas immediate– 5.00*Temporal Composite – 0.115*Animal Fluency – 0.256*MMSE + 0.336* Adas delayed

Supplement 2C*. Best model for single-step analysis with MRI*

**Supplementary Table 1**

|  | **ADNI (n=612)** | **BioFINDER-1 (n=392)** | **Comparison** |
| --- | --- | --- | --- |
| **Age years (SD)** | 72.9 (6.8) | 70.9 (5.5) | p<0.001 |
| **Education years (SD)** | 16.3 (2.7) | 11.8 (3.5) | p<0.001 |
| **Sex** | 274 female, 338 male | 190 female, 202 male | p=0.27 |
| **Progressing to dementia within 4 years (%)** | 301 (49.2%) | 141 (36.0%) | p<0.001 |
| **Progressing to dementia within 1 year (%)** | 122 (19.9%) | 15 (3.8%) | p<0.001 |
| **SCD & MCI** | SCD 110, MCI 502 | SCD 176, MCI 216 | p<0.001 |

*Supplementary Table 1. Comparison of included participants in ADNI and BioFINDER-1 at baseline. Comparison between age and education are calculated with t-test, sex, progression to dementia and SCD/MCI are calculated with Fisher exact test. Abbreviations: NC: non-progressor, SCD: subjective cognitive decline, MCI: mild cognitive impairment*

**Supplementary Table 2**

|  | **One-year progressors in ADNI** | | |
| --- | --- | --- | --- |
|  | **Low risk (N=5)** | **Intermediate risk (N=40)** | **High risk (N=77)** |
| **ADAS immediate recall, mean errors** | 3.0 (1.0) | 4.9 (1.2) | 6.9 (1.2) |
| **ADAS delayed recall, errors** | 3.0 (1.7) | 5.9 (1.8) | 8.4 (1.5) |
| **TMT A, sec** | 34.2 (9.3) | 40.8 (13.5) | 56.0 (26.5) |
| **TMT B, sec** | 93.2 (45.5) | 103.3(40.8) | 188.7 (85.4) |
| **MMSE (SD)** | 29.8 (0.4) | 28.0 (1.6) | 26.1 (1.6) |
| **Animal Fluency, no of words** | 22.2 (4.8) | 16.8 (3.6) | 12.4 (3.6) |

*Supplementary table 2. Table shows mean (SD) cognitive test results at baseline for individuals converting within a year depending on stratified as low, intermediate or high risk at the first step of the model.*

**Supplementary Table 3**

|  | **Total cohort in ADNI** | | |
| --- | --- | --- | --- |
|  | **Low risk (N=204)** | **Intermediate risk (N=226)** | **High risk (N=182)** |
| **ADAS immediate recall, mean errors** | 2.9 (1.1) | 4.9 (1.2) | 6.6 (1.2) |
| **ADAS delayed recall, errors** | 2.2 (1.3) | 5.4 (1.8) | 8.1 (1.7) |
| **TMT A, sec** | 30.7 (9.6) | 38.7 (14.4) | 53.4 (26.5) |
| **TMT B, sec** | 70.8 (26.6) | 96.7 (43.2) | 175.9 (79.4) |
| **MMSE (SD)** | 29.3 (0.8) | 28.0 (1.5) | 26.2 (1.7) |
| **Animal Fluency, no of words** | 22.1 (4.8) | 17.5 (4.0) | 13.5 (4.2) |

*Supplementary table 3. Table shows mean (SD) cognitive test results at baseline for individuals converting within a year depending on stratified as low, intermediate or high risk at the first step of the model.*

**Supplementary Table 4**

|  | **BioFINDER-1** | | | |
| --- | --- | --- | --- | --- |
| **Follow-up diagnosis in those who progressed to dementia** | Total included cohort | Screened as high risk at step 1 | Screened as high risk at step 2 | Screened as high risk in total model (% all progressions to this subtype) |
| **AD** | 92 | 50 | 22 | 77 (83.7%) |
| **VaD** | 22 | 12 | 5 | 17 (77.3%) |
| **DLB** | 6 | 2 | 2 | 4 (66.7%) |
| **bvFTD** | 6 | 4 | 0 | 4 (66.7%) |
| **Undetermined neurodegenerative disorder** | 4 | 3 | 0 | 3 (75%) |
| **PDD** | 4 | 1 | 1 | 2 (50%) |
| **NPH** | 2 | 0 | 1 | 1 (50%) |
| **svPPA** | 2 | 0 | 0 | 0 |
| **CBS** | 1 | 0 | 0 | 0 |
| **MSA** | 1 | 0 | 0 | 0 |
| **PSP** | 1 | 0 | 0 | 0 |

*Supplementary Table 4. Table shows diagnosis in individuals progressing to dementia within 4 years from BioFINDER-1. Abbreviations: AD = Alzheimer disease, VaD = Vascular Dementia, DLB = dementia with Lewy bodies, bvFTD = behavioral variant of frontotemporal dementia, PDD = Parkinson disease dementia, NPH = Normal pressure hydrocephalus, svPPA = semantic variant of primary progressive aphasia, CBS = corticobasal syndrome, MSA = Multiple system atrophy, PSP = progressive supranuclear palsy.*

**Supplementary Table 5: Two-step model trained in ADNI replicated in BioFINDER-1 including subjects who are followed** ≥**6 respectively** ≥**8 years or progress before this**

|  |  | **BioFINDER followed *≥*6 years** | | | **BioFINDER followed *≥*8 years** | | |
| --- | --- | --- | --- | --- | --- | --- | --- |
|  |  | **Progressing** | **Not progressing** | **Total** | **Progressing** | **Not progressing** | **Total** |
| Step 1 | High risk | 85 | 15 | 100 | 96 | 3 | 99 |
|  | Low risk | 7 | 99 | 106 | 12 | 58 | 70 |
|  | Total | 92 | 114 | 206 | 108 | 61 | 169 |
|  | Intermediate risk | 92 | 68 | 160 | 106 | 25 | 131 |
| Step 2 | High risk | 41 | 22 | 63 | 47 | 6 | 53 |
|  | Low risk | 19 | 35 | 54 | 23 | 14 | 37 |
|  | Total | 60 | 57 | 117 | 70 | 20 | 90 |
| Combined steps | High risk | 126 | 37 | 163 | 143 | 9 | 152 |
|  | Low risk | 26 | 134 | 160 | 35 | 72 | 107 |
|  | Total | 152 | 171 | 323 | 178 | 81 | 259 |

*Supplementary Table 5: Number of individuals progressing versus not progressing to dementia in the follow-up in BioFINDER-1 including subjects who are followed for ≥6 years or progressed before versus ≥8 years or progressed before. from the two-step model in ADNI*

**Supplementary Table 6: Two-step model trained in ADNI replicated in BioFINDER-1 including subjects who are followed** ≥**6 respectively** ≥**8 years or progress before this**

|  | **BioFINDER-1 followed *≥*6 years** | | | **BioFINDER-1 followed *≥*8 years** | | |
| --- | --- | --- | --- | --- | --- | --- |
|  | **Step 1** | **Step 2** | **Combined steps** | **Step 1** | **Step 2** | **Combined steps** |
| Prevalence of dementia progression | 44.7% | 51.3% | 47.1% | 63.9% | 77.8% | 68.7% |
| Sensitivity | 92.4% | 68.3% | 82.9% | 88.9% | 67.1% | 80.3% |
| Specificity | 86.8% | 61.4% | 78.4% | 95.1% | 70.0% | 88.9% |
| PPV | 85.0% | 65.1% | 77.3% | 97.0% | 88.7% | 94.1% |
| NPV | 93.4% | 64.8% | 83.8% | 82.9% | 37.8% | 67.3% |
| Accuracy | 89.3% | 65.0% | 80.5% | 91.1% | 67.8% | 83.0% |

*Supplementary Table 6: Table showing how well the 4-year prediction model trained in ADNI is when replicating in BioFINDER-participants who are followed for ≥6 years or progressed before versus ≥8 years or progressed before*

**Supplementary Table 7**

|  | **ADNI** | **BioFINDER-1** |
| --- | --- | --- |
| **Prevalence of dementia progression** | 49.2% | 36.0% |
| **Sensitivity** | 81.7% | 87.2% |
| **Specificity** | 84.6% | 67.3% |
| **PPV** | 83.7% | 60.0% |
| **NPV** | 82.7% | 90.4% |
| **Accuracy** | 83.2% | 74.5% |

Supplementary Table 7. Table showing *levels calculated from of individuals progressing versus not progressing to dementia within 4 years in the groups at a cutoff at 0.5 (probability threshold = 0.5) in ADNI and BioFINDER-1*

**Supplementary Table 8: Single-step analysis with MRI**

| **Study** | **ADNI** | | | **BioFINDER-1** | | |
| --- | --- | --- | --- | --- | --- | --- |
|  | Progressing | Not progressing | Total | Progressing | Not progressing | Total |
| High risk | 198 | 48 | 246 | 123 | 123 | 246 |
| Low risk | 21 | 201 | 222 | 3 | 111 | 114 |
| Total | 219 | 249 | 468 | 126 | 234 | 360 |

*Supplementary Table 8. Showing number of individuals progressing versus not progressing to dementia within 4 years in the groups at the at the >90% sensitivity level in ADNI and BioFINDER-1 including temporal volume in the model.*

**Supplementary Table 9: Single-step analysis with MRI**

| **Study** | **ADNI** | **BioFINDER-1** |
| --- | --- | --- |
| Prevalence of dementia progression | 46.8% | 35.0% |
| Sensitivity | 90.4% | 97.6% |
| Specificity | 80.7% | 47.4% |
| PPV | 80.5% | 50.0% |
| NPV | 90.5% | 97.4% |
| Accuracy | 85.3% | 65.0% |

*Supplementary Table 9. Showing levels calculated from of individuals progressing versus not progressing to dementia within 4 years in the groups at the >90% sensitivity level in ADNI and BioFINDER-1 including temporal volume in the model.*

**Supplementary figure 1: Figure showing progression to dementia using a one-step MRI-model**

**

*Supplementary figure 1: Showing individuals progressing to dementia in the training study ADNI (1A) and replicated in BioFINDER-1 (1B). This analysis included 468 individuals from ADNI. In this analysis, we found a model including the cognitive tests ADAS delayed and ADAS immediate, Animal Fluency including age, sex and the temporal composite. B: Replicated model in BioFINDER-1 (N=360).*
