# Supplementary material for: Individualized, cross‐validated prediction of future dementia using cognitive assessments in people with mild cognitive symptoms
Source: Alzheimers Dement. 2024 Oct 17;20(12):8625–38. doi: 10.1002/alz.14305 (PMC11667515; doi:10.1002/alz.14305)
Supplement: Supplementary file 2 — Supporting Information [file ALZ-20-8625-s002.docx]

**Supplementary figure 1: Figure showing progression to dementia using a one-step MRI-model**

**

*Supplementary figure 1: Showing individuals progressing to dementia in the training study ADNI (1A) and replicated in BioFINDER-1 (1B). This analysis included 468 individuals from ADNI. In this analysis, we found a model including the cognitive tests ADAS delayed and ADAS immediate, Animal Fluency including age, sex and the temporal composite. B: Replicated model in BioFINDER-1 (N=360).*
