# Supplementary material for: Individualized, cross‐validated prediction of future dementia using cognitive assessments in people with mild cognitive symptoms
Source: Alzheimers Dement. 2024 Oct 17;20(12):8625–38. doi: 10.1002/alz.14305 (PMC11667515; doi:10.1002/alz.14305)
Supplement: Supplementary file 3 — Supporting Information [file ALZ-20-8625-s003.docx]

**Supplementary Table 1**

|  | **ADNI (n=612)** | **BioFINDER-1 (n=392)** | **Comparison** |
| --- | --- | --- | --- |
| **Age years (SD)** | 72.9 (6.8) | 70.9 (5.5) | p<0.001 |
| **Education years (SD)** | 16.3 (2.7) | 11.8 (3.5) | p<0.001 |
| **Sex** | 274 female, 338 male | 190 female, 202 male | p=0.27 |
| **Progressing to dementia within 4 years (%)** | 301 (49.2%) | 141 (36.0%) | p<0.001 |
| **Progressing to dementia within 1 year (%)** | 122 (19.9%) | 15 (3.8%) | p<0.001 |
| **SCD & MCI** | SCD 110, MCI 502 | SCD 176, MCI 216 | p<0.001 |

*Supplementary Table 1. Comparison of included participants in ADNI and BioFINDER-1 at baseline. Comparison between age and education are calculated with t-test, sex, progression to dementia and SCD/MCI are calculated with Fisher exact test. Abbreviations: NC: non-progressor, SCD: subjective cognitive decline, MCI: mild cognitive impairment*

**Supplementary Table 2**

|  | **One-year progressors in ADNI** | | |
| --- | --- | --- | --- |
|  | **Low risk (N=5)** | **Intermediate risk (N=40)** | **High risk (N=77)** |
| **ADAS immediate recall, mean errors** | 3.0 (1.0) | 4.9 (1.2) | 6.9 (1.2) |
| **ADAS delayed recall, errors** | 3.0 (1.7) | 5.9 (1.8) | 8.4 (1.5) |
| **TMT A, sec** | 34.2 (9.3) | 40.8 (13.5) | 56.0 (26.5) |
| **TMT B, sec** | 93.2 (45.5) | 103.3(40.8) | 188.7 (85.4) |
| **MMSE (SD)** | 29.8 (0.4) | 28.0 (1.6) | 26.1 (1.6) |
| **Animal Fluency, no of words** | 22.2 (4.8) | 16.8 (3.6) | 12.4 (3.6) |

*Supplementary table 2. Table shows mean (SD) cognitive test results at baseline for individuals converting within a year depending on stratified as low, intermediate or high risk at the first step of the model.*

**Supplementary Table 3**

|  | **Total cohort in ADNI** | | |
| --- | --- | --- | --- |
|  | **Low risk (N=204)** | **Intermediate risk (N=226)** | **High risk (N=182)** |
| **ADAS immediate recall, mean errors** | 2.9 (1.1) | 4.9 (1.2) | 6.6 (1.2) |
| **ADAS delayed recall, errors** | 2.2 (1.3) | 5.4 (1.8) | 8.1 (1.7) |
| **TMT A, sec** | 30.7 (9.6) | 38.7 (14.4) | 53.4 (26.5) |
| **TMT B, sec** | 70.8 (26.6) | 96.7 (43.2) | 175.9 (79.4) |
| **MMSE (SD)** | 29.3 (0.8) | 28.0 (1.5) | 26.2 (1.7) |
| **Animal Fluency, no of words** | 22.1 (4.8) | 17.5 (4.0) | 13.5 (4.2) |

*Supplementary table 3. Table shows mean (SD) cognitive test results at baseline for individuals converting within a year depending on stratified as low, intermediate or high risk at the first step of the model.*

**Supplementary Table 4**

|  | **BioFINDER-1** | | | |
| --- | --- | --- | --- | --- |
| **Follow-up diagnosis in those who progressed to dementia** | Total included cohort | Screened as high risk at step 1 | Screened as high risk at step 2 | Screened as high risk in total model (% all progressions to this subtype) |
| **AD** | 92 | 50 | 22 | 77 (83.7%) |
| **VaD** | 22 | 12 | 5 | 17 (77.3%) |
| **DLB** | 6 | 2 | 2 | 4 (66.7%) |
| **bvFTD** | 6 | 4 | 0 | 4 (66.7%) |
| **Undetermined neurodegenerative disorder** | 4 | 3 | 0 | 3 (75%) |
| **PDD** | 4 | 1 | 1 | 2 (50%) |
| **NPH** | 2 | 0 | 1 | 1 (50%) |
| **svPPA** | 2 | 0 | 0 | 0 |
| **CBS** | 1 | 0 | 0 | 0 |
| **MSA** | 1 | 0 | 0 | 0 |
| **PSP** | 1 | 0 | 0 | 0 |

*Supplementary Table 4. Table shows diagnosis in individuals progressing to dementia within 4 years from BioFINDER-1. Abbreviations: AD = Alzheimer disease, VaD = Vascular Dementia, DLB = dementia with Lewy bodies, bvFTD = behavioral variant of frontotemporal dementia, PDD = Parkinson disease dementia, NPH = Normal pressure hydrocephalus, svPPA = semantic variant of primary progressive aphasia, CBS = corticobasal syndrome, MSA = Multiple system atrophy, PSP = progressive supranuclear palsy.*

**Supplementary Table 5: Two-step model trained in ADNI replicated in BioFINDER-1 including subjects who are followed** ≥**6 respectively** ≥**8 years or progress before this**

|  |  | **BioFINDER followed *≥*6 years** | | | **BioFINDER followed *≥*8 years** | | |
| --- | --- | --- | --- | --- | --- | --- | --- |
|  |  | **Progressing** | **Not progressing** | **Total** | **Progressing** | **Not progressing** | **Total** |
| Step 1 | High risk | 85 | 15 | 100 | 96 | 3 | 99 |
|  | Low risk | 7 | 99 | 106 | 12 | 58 | 70 |
|  | Total | 92 | 114 | 206 | 108 | 61 | 169 |
|  | Intermediate risk | 92 | 68 | 160 | 106 | 25 | 131 |
| Step 2 | High risk | 41 | 22 | 63 | 47 | 6 | 53 |
|  | Low risk | 19 | 35 | 54 | 23 | 14 | 37 |
|  | Total | 60 | 57 | 117 | 70 | 20 | 90 |
| Combined steps | High risk | 126 | 37 | 163 | 143 | 9 | 152 |
|  | Low risk | 26 | 134 | 160 | 35 | 72 | 107 |
|  | Total | 152 | 171 | 323 | 178 | 81 | 259 |

*Supplementary Table 5: Number of individuals progressing versus not progressing to dementia in the follow-up in BioFINDER-1 including subjects who are followed for ≥6 years or progressed before versus ≥8 years or progressed before. from the two-step model in ADNI*

**Supplementary Table 6: Two-step model trained in ADNI replicated in BioFINDER-1 including subjects who are followed** ≥**6 respectively** ≥**8 years or progress before this**

|  | **BioFINDER-1 followed *≥*6 years** | | | **BioFINDER-1 followed *≥*8 years** | | |
| --- | --- | --- | --- | --- | --- | --- |
|  | **Step 1** | **Step 2** | **Combined steps** | **Step 1** | **Step 2** | **Combined steps** |
| Prevalence of dementia progression | 44.7% | 51.3% | 47.1% | 63.9% | 77.8% | 68.7% |
| Sensitivity | 92.4% | 68.3% | 82.9% | 88.9% | 67.1% | 80.3% |
| Specificity | 86.8% | 61.4% | 78.4% | 95.1% | 70.0% | 88.9% |
| PPV | 85.0% | 65.1% | 77.3% | 97.0% | 88.7% | 94.1% |
| NPV | 93.4% | 64.8% | 83.8% | 82.9% | 37.8% | 67.3% |
| Accuracy | 89.3% | 65.0% | 80.5% | 91.1% | 67.8% | 83.0% |

*Supplementary Table 6: Table showing how well the 4-year prediction model trained in ADNI is when replicating in BioFINDER-participants who are followed for ≥6 years or progressed before versus ≥8 years or progressed before*

**Supplementary Table 7**

|  | **ADNI** | **BioFINDER-1** |
| --- | --- | --- |
| **Prevalence of dementia progression** | 49.2% | 36.0% |
| **Sensitivity** | 81.7% | 87.2% |
| **Specificity** | 84.6% | 67.3% |
| **PPV** | 83.7% | 60.0% |
| **NPV** | 82.7% | 90.4% |
| **Accuracy** | 83.2% | 74.5% |

Supplementary Table 7. Table showing *levels calculated from of individuals progressing versus not progressing to dementia within 4 years in the groups at a cutoff at 0.5 (probability threshold = 0.5) in ADNI and BioFINDER-1*

**Supplementary Table 8: Single-step analysis with MRI**

| **Study** | **ADNI** | | | **BioFINDER-1** | | |
| --- | --- | --- | --- | --- | --- | --- |
|  | Progressing | Not progressing | Total | Progressing | Not progressing | Total |
| High risk | 198 | 48 | 246 | 123 | 123 | 246 |
| Low risk | 21 | 201 | 222 | 3 | 111 | 114 |
| Total | 219 | 249 | 468 | 126 | 234 | 360 |

*Supplementary Table 8. Showing number of individuals progressing versus not progressing to dementia within 4 years in the groups at the at the >90% sensitivity level in ADNI and BioFINDER-1 including temporal volume in the model.*

**Supplementary Table 9: Single-step analysis with MRI**

| **Study** | **ADNI** | **BioFINDER-1** |
| --- | --- | --- |
| Prevalence of dementia progression | 46.8% | 35.0% |
| Sensitivity | 90.4% | 97.6% |
| Specificity | 80.7% | 47.4% |
| PPV | 80.5% | 50.0% |
| NPV | 90.5% | 97.4% |
| Accuracy | 85.3% | 65.0% |

*Supplementary Table 9. Showing levels calculated from of individuals progressing versus not progressing to dementia within 4 years in the groups at the >90% sensitivity level in ADNI and BioFINDER-1 including temporal volume in the model.*
